# A gender specific improved survival related to stromal miR-143 and miR-145 expression in non-small cell lung cancer

**DOI:** 10.1038/s41598-018-26864-w

**Published:** 2018-06-04

**Authors:** Kaja Skjefstad, Charles Johannessen, Thea Grindstad, Thomas Kilvaer, Erna-Elise Paulsen, Mona Pedersen, Tom Donnem, Sigve Andersen, Roy Bremnes, Elin Richardsen, Samer Al-Saad, Lill-Tove Busund

**Affiliations:** 1 Department of Medical Biology, UiT The Arctic University of Norway, Mailbox 6050 Langnes, N-9037 Tromso, Norway; 2 Department of Clinical Medicine, UiT The Arctic University of Norway, Mailbox 6050 Langnes, N-9037 Tromso, Norway; 3 Department of Oncology, University Hospital of North Norway, Mailbox 13, N-9038 Tromso, Norway; 4 Department of Clinical Pathology, University Hospital of North Norway, Mailbox 46, N-9038 Tromso, Norway

## Abstract

Micro RNAs (miRNA) are small non-coding RNAs that post-transcriptionally regulate gene expression. Dysregulation of miRNA cluster 143/145 has been reported in several malignancies, but their role in non-small cell lung cancer (NSCLC) remains elusive. This study investigates the prognostic impact of miR-143 and miR-145 in primary tumors and metastatic lymph nodes in NSCLC tissue. Tissue from 553 primary tumors and 143 matched metastatic lymph nodes were collected and tissue microarrays were constructed. *In situ* hybridization was used to evaluate miR-143 and miR-145 expression in tumor epithelial cells and stromal cells in the primary tumors and lymph nodes. *In vivo* data was supplemented with functional studies of cell lines *in vitro* to evaluate the role of miR-143 and miR-145 in NSCLC tumorigenesis. In our cohort, stromal miR-143 (S-miR-143) and miR-145 (S-miR-145) expression in primary tumor tissue were independent prognosticators of improved disease-specific survival (DSS) in female (S-miR-143, HR: 0.53, p = 0.019) and male patients (S-miR-145, HR: 0.58, p = 0.021), respectively. Interesting correlations between the miR cluster 143/145 and previously investigated steroid hormone receptors from the same cohort were identified, substantiating their gender dependent significance.

## Introduction

Lung cancer remains the leading cancer killer in the world with more than 1.6 million estimated annual deaths, worldwide^1^. The predominant histological subtype, non-small cell lung cancer (NSCLC), accounts for 85% of cases and can be further divided into subgroups according to the recent WHO classification; the most frequent being adenocarcinoma and squamous cell carcinoma^2^. Surgical resection is the main curative treatment modality for NSCLC, but unfortunately, the majority of patients are diagnosed in advanced stages and thus not eligible for surgery. Despite development in surgical techniques, diagnostic technologies and the implementation of biologic treatment including immunotherapy, the 5-year survival remains depressing at only 18%^3^. To optimize therapy and improve the overall survival, it is pivotal to uncover better prognostic and predictive molecular markers.

microRNAs (miRNAs) are small non-coding RNA elements important in various biological processes, including tumorigenesis^4^. They negatively regulate protein translation by binding to the 3′UTR of target messenger RNAs (mRNAs) leading to mRNA degradation or suppression of translation^5^. miRNA expression correlates with biological and clinical characteristics of tumors; differentiation, aggression, tissue type and therapy response^6^. Further, “miRNA replacement therapy” provides a novel treatment opportunity by reintroducing downregulated miRNA into cancer cells^7^. A phase I clinical trial of miRNA replacement therapy in thoracic cancers, based on the miR-15/107 group of miRNAs, was recently completed with promising results^8^.

miR cluster 143/145 consists of two miRNAs, miR-143 and miR-145, transcribed from a gene cluster on chromosome 5. It regulates multiple genes involved in cancer cell growth, including well-established cancer related hormone receptors such as ERα, and is generally regarded as a tumor suppressor^9–11^. Reports have indicated a possible prognostic role in non-small cell lung cancer^12,13^. The presented study investigates the prevalence and prognostic significance of miR-143 and miR-145 in NSCLC. The utilization of *in situ* hybridization allow both localization of expression according to cell-type and sub-cellular compartment. Further, correlations with steroid hormone receptors progesterone receptor (PR), estrogen receptor alpha (ERα), estrogen receptor beta (ERβ) and aromatase enzyme (AR), previously investigated by our group, were explored. The clinicopathological findings were supplied with data from functional *in vitro* studies.

## Materials and Methods

### Patients

NSCLC patients who underwent radical resection at the Nordland Central Hospital and the University Hospital of North Norway from 1990 to 2011, were retrospectively included in this study. Six-hundred-and-thirty-three patients were identified from the hospital records. Of these, 80 patients were excluded due to (1) inadequate fixation of paraffin-embedded tissue blocks (n = 26), (2) radiotherapy or chemotherapy prior to surgery (n = 15), (3) other malignancy within 5 years ahead of an NSCLC diagnosis (n = 39), leaving 553 patients eligible for inclusion. One-hundred-and-seventy-two of the included patients had confirmed metastatic lymph node tissue disease (LN+). Of these, 143 patients had lymph node specimens available for analysis. The eight edition of the International Union Against Cancer TNM classification was used to re-stage all patients, and the tumors were histologically re-classified according to the 2015 World Health Organization Classification of Lung Tumors^2,14^. Follow-up data as of October 1^st^ 2013.

### Tissue microarray construction

All specimens were embedded in paraffin blocks and examined by two experienced pathologists. Detailed methodology regarding TMA construction has previously been published^15^. Briefly, (1) representative areas of stromal and tumor tissue in primary tumors and tumor tissue from lymph nodes were identified and sampled with a 0.6 mm stylet, (2) transferred to the recipient TMA block and (3) cut into 4μm sections with a Micron microtome (HM355S) prior to *in situ* hybridization. Normal lung tissue far from the site of the tumor, and lung tissue samples from 20 emphysema patients without any history of neoplastic disease, were used as controls and for comparing biomarker expression level in malignant vs non-malignant tissue.

### *In situ* hybridization (ISH)

miR-143 and miR-145 expression was analyzed by *in situ* hybridization (ISH) using the Ventana Discovery Ultra (Ventana Medical Inc, Arizona, USA). Optimization of biomarker detection included: RNA degradation prevention, testing of reagent concentration for the tissue of interest and detection method, and testing of hybridization temperatures for each probe with RNA Tm (melting temperature) as guideline. Digoxigenin (DIG) labeled lock nucleic acid (LNA) probes for miR-145-5p (hsa-miR-145, Prod. No. 88068-15, concentration: 2.5 nM), miR-143-3p (hsa-miR-143, Prod. No. 38515-15, concentration: 10 nM), negative control (Scramble miR, Prod. No. 99004-15, concentration: 10 nM) and positive control (U6 has/mmu/rno, Prod. No. 99002-15, concentration: 0.5 nM) were used in this study. Exiqon validated the LNA^TM^ miR probes by CE (Capillary Electrophoresis) or HPLC (High-Performance Liquid Chromotography) and confirmed identity of compound by MS (Mass Spectrometry). A TMA multi organ block was used as positive and negative tissue controls.

4 µm TMA sections were incubated overnight at 60 °C to attach tissue to Super Frost Plus slides. To ensure good distribution of reagents and protect sections from desiccation, LCS (Liquid Coverslip oil, Roche, 5264839001) was added. Deparaffinization was performed in EZ Prep buffer (Roche 5279755001) at 68 °C (3 × 12 min). Demasking was done at 95 °C with CC1 buffer (Roche, 6414575001) for 40 minutes. Subsequently, sections were rinsed with Reaction Buffer (Roche 5353955001) and RiboWash, SSPE buffer (Roche 5266262001).

All slides were denaturated for 8 min. at 90 °C. Hybridization with probes was performed for 60 min at 54 °C for miR-145, 55 °C for miR-143, 57 °C for scramble miR and 55 °C for U6. Stringent wash procedures were done at 2 × 8 min with 2.0X RiboWash, SSPE buffer with the same temperatures as used under hybridization for each probe. Blocking against unspecific bindings followed, with blocking solution (Roche, 5268869001) for 16 min. at 37 °C. Alkaline phosphatase (AP)-conjugated anti DIG (Anti-DIG-AP Multimer, Roche 07256302001) was incubated for 20 min. at 37 °C for immunologic detection. After rinsing, substrate enzymatic reactions were carried out with NBT/BCIP (CromoMap Blue kit, Roche 526661001) for 60 min at 37 °C, to give a blue precipitate to detect the microRNA. Sections were again rinsed and counterstained in 4 min with Red Stain II (Roche 5272017001). Increasing gradients of ethanol solutions was used for dehydration. Finally, all sections were mounted using the Histokitt mounting medium (Assistant-Histokitt, 1025/250 Sondheim/Rhoen Germany).

### Scoring of ISH

All tissue samples were independently and semi-quantitatively scored by an experienced pathologist (SAS) and a trained medical doctor (KS). Biomarkers were evaluated by intensity in neoplastic epithelial cells and stromal cells; 0 (no staining), 1 (weak), 2 (intermediate) and 3 (strong) and density in stromal cells; 0 = absent, 1 = 1–5%, 2 = 6–50%, 3 = >50%. Due to homogenous staining in neoplastic epithelial cells, scoring of biomarker density was not deemed necessary. For stromal biomarker expression (S-miR) the mean value of intensity and density combined, was calculated. Staining of fibroblasts, fibrocytes, lymphocytes, smooth muscle cells (SMC) and endothelial cells in blood and lymph vessels were included while scoring tumor stroma. Striking positivity was noted in endothelial cells lining the blood vessels and SMCs, including the smallest capillaries. Each variable was dichotomized for survival analyses based on a minimal p-value approach. A high score was defined as a score ≥ mean value for stromal-miR-143 (S-miR-143, mean value: 1.87) and tumor-miR-143 (T-miR-143, mean value: 1.98) and >0 for S-miR-145 and T-miR-145. The same scoring approach was used in PT, LN+, positive and negative tissue controls. For LN+ however, the stromal compartment was not scored due to large numbers of excessively stained lymphocytes. In normal lung tissue from NSCLC patients, collected far from the site of the tumor, miR-143 was prominently expressed in type 2 pneumocytes and macrophages. Collagen and endothelial cells lining the alveolar wall were mostly negative. miR-145 expression was observed in a few pneumocytes type I, while most were negative. Staining in macrophages was predominantly negative.

### Functional studies

#### Cell cultures

Four lung cancer cell lines were used: the adenocarcinoma cell line A549 (ATCC® CCL-185™), the squamous cell carcinoma cell line H520 (ATCC® HTB182™), and the two large cell carcinoma cell lines H460 (ATCC ® HTB-177™) and H661 (ATCC® HTB183™). All cells were cultured in RPMI-1640 media (# R8758, Sigma-Aldrich, St. Louis, USA) supplemented with 10% fetal bovine serum (# S0415, Biochrom, Berlin, Germany) and 1x penicillin-streptomycin antibiotic mixture (# P0781, Sigma-Aldrich, St. Louis, USA) and incubated at 37 °C in 5% CO_2_ humidified atmosphere.

#### Cell transfection

Cells were transiently transfected with either 100 nM has-miR-143-3p Pre-miR™ miRNA Precursor (catalog# PM10883, Thermo Fisher Scientific, USA) and/or 100 nM has-miR-145-5p Pre-miR™ miRNA Precursor (catalog# PM11480, Thermo Fisher Scientific, USA), alongside the Cy3™ Dye-Labeled Pre-miR Negative Control #1 (catalog# AM17120, Thermo Fisher Scientific, USA) using the transfection reagent Lipofectamine® 2000 (catalog#11668-019, Life Technologies, Waltham, USA). Transfected Cy3™ Dye-Labeled Pre-miR Negative Control emits fluorescent light when exposed to UV-light, and using a fluorescence microscope, the transfection efficiency was evaluated to be 80–95%.

### Total RNA isolation

Total RNA from the cells were isolated using the miRNeasy Mini Kit (cat.# 217004, Qiagen, Hilden, Germany). First, 700 μl QIAzol lysis reagent was used to lyse the cells before homogenization and a 5 minute incubation at room temperature. Second, 140 μl chloroform was added, samples shaken, and then incubated for 3 minutes at room temperature. Third, samples were centrifuged at 12000 G for 15 minutes at 4 °C before the upper aqueous phase was transferred and mixed with 100% ethanol. Finally, the samples were transferred to the RNeasy® Mini column and washed in several steps before elution with 50 μl ddH2O. Samples were stored at −70 °C.

### cDNA synthesis

For the first strand cDNA synthesis, the miScript II RT Kit (cat.# 218160, Qiagen, Hilden, Germany) was used. First, 100 ng total RNA was mixed with 4 μl 5X miScript HiSpec buffer, 2 μl 10X Nucleics mix, 2 μl miScript reverse transcriptase mix, and RNase-free water to a final volume of 20 μl. Second, samples were incubated for 1 hour at 37 °C, and then incubated at 95 °C for 5 minutes. Finally, all samples were diluted to a total volume of 200 μl using RNase-free water, and stored at −70 °C.

### RT-PCR

Endogenous levels of miR-143 and miR-145 in the cancer cells were quantified relative to the non-cancerous lung cell line NL20 (ATCC® CRL-2503™), and normalized to the stably expressed reference snRNA RNU6 using real-time PCR and the miScript SYBR® Green PCR Kit (catalog# 218073, Qiagen, Hilden, Germany). Primers were miScript Primer Assays Hs_miR-143_1 miScript Primer Assay (catalog# MS00003514, Qiagen, Hilden, Germany), Hs_miR-145_1 miScript Primer Assay (catalog# MS00003528, Qiagen, Hilden, Germany) and Hs_RNU6-2_11 miScript Primer Assay (catalog# MS00033740, Qiagen, Hilden, Germany), according to the manufacturers protocol. In short, a total volume of 25 µl/well in a 96-well plate included 1 µl cDNA mixed with 12.5 µl 2x QuantiTect SYBR Green PCR Master Mix, 2.5 µl 10x miScript Universal Primer, 2.5 µl 10x miScript Primer Assay, and 6.5 µl RNase-free Water. The plate was sealed and centrifuged for 1 minute at 1000 G before it was placed in the 7300 Real-Time PCR System (Thermo Fisher Scientific, Waltham, Massachusetts, USA). Each sample was analyzed in quadruplicates, and two independent experiments were performed.

### Proliferation assay

The ability of cancer cells to proliferate was evaluated using the real-time cell analyzer xCELLigence, RTCA DP (catalog#05469759001, ACEA Biosciences, San Diego, USA) fitted with the E-plate 16 (catalog#05469830001, ACEA Biosciences, San Diego, USA). Prior to seeding, cells were trypsinized until detached, resuspended in complete growth media, and counted. In accordance with the manufacturer protocol, cells were seeded in quadruplicates into an E-plate after baseline measurements. The E-plate containing cells was positioned in the RTCA DP instrument, located in an incubator preserving the same conditions as used for routine cultivation of cell lines. The cell index was automatically measured every 30 minutes throughout the experiment duration. Growth curves were calculated with the RTCA software version 1.2.1. A minimum of three independent experiments were performed for each cell line.

### Migration assay

The ability of cancer cells to migrate was assessed using ibidi^TM^ culture inserts (ibidi GmbH, Planegg, Germany). The inserts consist of two 0.22 cm^2^ silicone chambers separated by a 0.5 mm divider. The inserts were positioned into a 12-well tissue culture dish, one insert per well. Roughly 70 µl pre-transfected cell-suspension containing 4–6 × 10^5^ cells/ml were added to each chamber. The cells were left to adhere for 24 hours before the insert was removed and images acquired across the cell-free zone at time points 0 hours and 20 hours. The migration potential into the 0.5 mm gap was calculated using the free online software TScratch, version 1.0 (CSElab, Computational Science and Engineering Laboratory, Switzerland). Initially, the functional experiments for this study were designed using three cell lines; the large cell carcinoma cell line H460, the squamous cell carcinoma cell line H520, and the adenocarcinoma cell line A549. In our experiments, however, the cell lines H460 and H520 did not exhibit migrational properties, leaving only the A549 cell line representing the migration experiment. To strengthen our results, we therefor included the large cell carcinoma cell line H661 in the migration study.

### Statistical methods

The statistical package IBM SPSS (version 24 IBM Corp., Armonk, NY USA) was used to perform all statistical analyses.

Interobserver reliability between scorers was assessed by a two-way random effects model with absolute agreement definition. Associations between marker expression, and marker expression and clinicopathological parameters, were examined by Spearman’s rank correlation and *χ*^2^ test or Fisher’s exact. Wilcoxon non-parametrical test was used to assess the difference in biomarker expression between lung tumor tissue and non-malignant lung tissue. Statistical significance between proliferation curves was assessed by one-way ANOVA. The Kaplan-Meier method was used to visualize association between marker expression and disease-specific survival (DSS) and the statistical significance between survival curves was tested using the log-rank test. DSS was defined as the time from surgery to lung cancer death. Variables with significant p-values from the univariate analyses were entered into Cox proportional Hazard models. The final models were derived from a backward conditional method with probability for stepwise entry and removal at 0,05 and 0,10.

### Ethics

The Regional Committee for Medical and Health Research Ethics (REK Nord), alongside the Norwegian Data Protection have approved this study (protocol ID: 2011/2503). Due to the retrospective study design, the majority of patients were diseased and the tissue specimens over 10 years old. Thus, written patient consent was not deemed necessary by REK Nord. All patients were anonymously included in the database. A trial number for each patient was used when pairing clinical information with the respective patients. Clinical information was reported according to the REMARK guidelines^16^. The authors confirm that all experiments were performed in accordance with relevant guidelines and regulations. The database buildup was approved by The Data Protection Official for Research (NSD).

## Results

### Patient characteristics

Clinical, histopathological and demographic variables and their impact on DSS are presented in Table 1. The median age was 67 years (range, 28–85), 373 patients (68%) were male, and the majority, 532 patients (96%), were current or previous smokers. The median follow-up time of survivors was 86 months (range, 34–267). Postoperative radiotherapy was administered to 76 (14%) patients due to non-radical surgical margins or nodal metastasis. Adjuvant chemotherapy was introduced in Norway in 2005, 43 (8%) patients received this treatment.

**Table 1:** Clinical and pathological variables as predictors of disease-specific survival (DSS) in NSCLC patients (univariate analyses; log-rank test; N = 553, 180 and 373, respectively).

	Overall cohort				Female patients				Male patients			
	N(%)	5 year DSS (%)	Median DSS (mo)	p	N(%)	5 year DSS (%)	Median DSS (mo)	p	N(%)	5 year DSS (%)	Median DSS(mo)	p
Age				0.656				0.637				0.827
≤65	234 (42.3)	58	127		77 (42.8)	62	190		157 (42.1)	56	98	
>65	319 (57.7)	58	NR		103 (57.2)	65	NR		216 (57.9)	44	88	
Sex				**0**.**025**								
Female	180 (32.5)	64	190									
Male	373 (67.5)	55	91									
ECOG perf. status				**0**.**009**				0.400				**0**.**020**
0	324 (58.6)	63	235		112 (62.2)	67	NR		212 (56.8)	60	235	
1	191 (34.5)	52	71		56 (31.1)	60	127		135 (36.2)	48	51	
2	38 (6.9)	52	NR		12 (6.7)	55	NR		26 (7.0)	50	NR	
Smoking				0.069				0.732				0.060
Never	21 (3.8)	50	21		11 (6.1)	64	189		10 (2.7)	33	18	
Present	350 (63.3)	62	235		115 (63.9)	67	NR		235 (63.0)	59	235	
Previous	182 (32.9)	52	84		54 (30.0)	58	NR		128 (34.3)	49	57	
Weightloss				0.971				0.603				0.637
<10%	498 (90.1)	58	190		163 (90.6)	63	190		335 (89.8)	56	91	
≥10%	55 (9.9)	59	NR		17 (9.4)	68	NR		38 (10.2)	54	98	
Surgical procedure				<**0**.**001**				**0**.**024**				<**0**.**001**
Wedge/Lobectomy	411 (74.3)	64	235		148 (82.2)	68	190		263 (70.5)	61	235	
Pulmonectomy	142 (25.7)	42	30		32 (17.8)	42	37		110 (29.5)	42	29	
Margins				0.105				0.088				0.431
Free	506 (91.5)	59	190		166 (92.2)	65	190		340 (91.2)	56	98	
Not free	47 (8.5)	47	57		14 (7.8)	51	64		33 (8.8)	45	47	
Tstage				<**0**.**001**				**0**.**009**				<**0**.**001**
1a	14 (2.5)	93	NR		5 (2.8)	100	NR		9 (2.4)	89	NR	
1b	71 (12.8)	79	NR		30 (16.7)	82	NR		41 (11.0)	77	NR	
1c	95 (17.2)	64	190		33 (18.3)	66	NR		62 (16.6)	63	235	
2a	135 (24.4)	57	88		35 (19.4)	65	NR		100 (26.8)	54	83	
2b	73 (13.2)	48	47		28 (15.6)	60	NR		45 (12.1)	40	40	
3	104 (18.8)	56	NR		36 (20.0)	60	NR		68 (18.2)	54	98	
4	61 (11.1)	31	21		13 (7.2)	23	NR		48 (12.9)	36	19	
Nstage				<**0**.**001**				<**0**.**001**				<**0**.**001**
0	379 (68.5)	70	235		132 (73.3)	74	NR		247 (66.2)	67	235	
1	118 (21.3)	36	35		23 (12.8)	42	47		95 (25.5)	35	27	
2	56 (10.2)	23	21		25 (13.9)	30	35		31 (8.3)	16	15	
Pathological stage				<**0**.**001**				<**0**.**001**				<**0**.**001**
I	232 (42.0)	74	235		78 (43.3)	81	NR		154 (41.3)	70	235	
II	185 (33.4)	59	114		61 (33.9)	66	NR		124 (33.2)	56	91	
IIIA + B	136 (24.6)	28	21		41(22.8)	29	36		95 (25.5)	27	17	
Histology				0.241				0.431				0.125
SQCC	307 (55.5)	64	235		77 (42.8)	71	NR		230 (61.7)	61	235	
ADC	239 (43.2)	52	73		100 (55.6)	59	190		139 (37.3)	46	57	
Other^a^	7 (1.3)	67	NR		3 (1.6)	50	11		4 (1.0)	75	NR	
Vascular infiltration				<**0**.**001**				**0**.**040**				<**0**.**001**
No	453 (82.0)	62	235		136 (75.6)	68	190		317 (85.0)	60	235	
Yes	97 (17.5)	38	35		42 (23.3)	49	47		55 (14.7)	25	22	
Missing	(0.5)				2 (1.1)				1 (0.3)			

### Scoring agreement

Scoring agreement between the scorers (SAS and KS) was excellent; ICC were 0.80 (p < 0.001) and 0.97 (p < 0.001) for miR-143 and miR-145, respectively.

### miR-143 and miR-145 expression in NSCLC cells

#### ISH expression of miR-143 and miR-145 in NSCLC cells and metastatic lymph nodes

**Figure 1:**
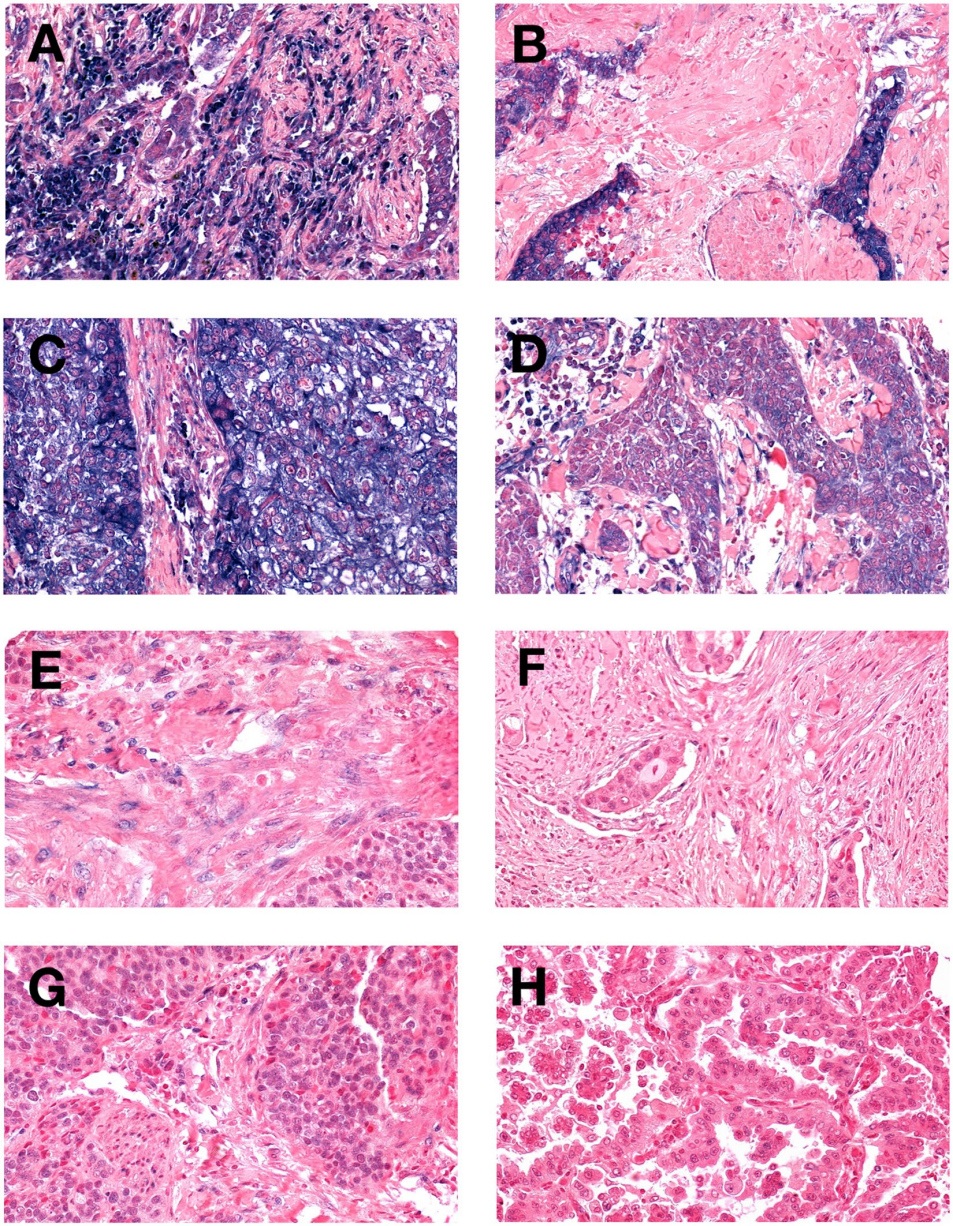
*In situ* hybridization staining of miR-143 and miR-145 in NSCLC. High miR-143 expression: Panel (A) stromal cells, Panel (C) tumor cells. Low miR-143 expression: Panel (B) stromal cells, Panel (D) cancer cells. High miR-145 expression: Panel (E) stromal cells, Panel (G) cancer cells. Low miR-145 expression: Panel (F) stromal cells, Panel (H) cancer cells. 400× magnification.

miR-143 was primarily observed in the cytoplasm of tumor epithelial and stromal cells, while miR-145 was mainly observed in the epithelial and stromal cell nuclei (Fig. 1). Table 2 reports miR-143 and miR-145 expression according to tissue compartment and gender. Neoplastic epithelial and stromal cells had significantly increased levels of miR-143 and miR-145 compared to non-malignant lung tissue (T-miR-143: p < 0.001, S-miR-143: p < 0.001, T-miR-145: p = 0.005, S-miR-145: p = 0.020). T-miR-143 expression in PT and LN+ was significantly correlated (0.220, p < 0.001). There was a significant correlation between miR-145 expression in neoplastic epithelial cells and stromal cells (0,362, p < 0.001). Similarly, miR-145 expression in PT and LN+ was significantly correlated (0,366, p < 0.001)

**Table 2:** Prognostic Effect of intraepithelial (T) and stromal (S) miR-143 and miR-145 expression in Primary Tumors on Disease-Specific Survival (Univariate Analyses; Log-Rank Test, N = 553, 180 and 373, respectively).

	Overall cohort				Female patients				Male patients			
	N (%)	5 year (%)	Median (mo)	p	N (%)	5 year (%)	Median (mo)	p	N (%)	5 year (%)	Median (mo)	p
S-miR-143				0.075				**0**.**011**				0.589
Low	261 (47.2)	55	104		88 (48.9)	55	127		173 (46.4)	55	104	
High	261 (47.2)	62	235		83 (46.1)	73	190		178 (47.7)	56	98	
Missing	31 (5.6)				9 (5.0)				22 (5.9)			
T-miR-143				0.071				0.699				0.160
Low	198 (35.7)	63	NR		64 (35.6)	65	NR		134 (35.9)	62	NR	
High	320 (58.0)	55	114		106 (58.9)	62	190		214 (57.4)	52	71	
Missing	35 (6.3)				10 (5.5)				25 (6.7)			
S-miR-145				0.130				0.602				**0**.**013**
Low	61 (11.1)	46	45		20 (11.1)	63	NR		41 (11.0)	38	32	
High	462 (83.5)	60	190		150 (83.3)	63	190		312 (83.6)	58	235	
Missing	30 (5.4)				10 (5.6)				20 (5.4)			
T-miR-145				0.111				0.068				0.592
Low	69 (12.5)	66	NR		26 (14.4)	76	NR		43 (11.5)	59	NR	
High	432 (78.1)	57	114		139 (77.2)	60	190		293 (78.6)	56	98	
Missing	52 (9.4)				15 (8.4)				37 (9.9)			
S-miR-143/S-miR-145				**0**.**007**				0.345				**0**.**004**
Low^a^	32 (5.8)	34	32		11 (6.1)	44	45		21 (5.6)	29	21	
High^b^	482 (87.2)	60	190		158 (87.8)	64	190		324 (86.9)	57	114	
Missing	39 (7.0)				11 (6.1)				28 (7.5)			

Note: Bold numbers indicate p < 0.05. Abbreviations: S-miR, stromal miR expression. T-miR, tumor epithelial expression. N, number. NR, not reached. Mo, months.

^a^Low: low S/low S.

^b^High: high S/high S, high S/low S, low S/high.

#### Relative expression of miR-143 and miR-145 in NSCLC cell lines

Endogenous levels of miR-143 and miR-145 in the studied NSCLC cell lines were quantified by qPCR, relative to the non-cancerous lung cell line NL20. Both miR-143 and miR-145 were downregulated in all the selected cell lines, compared to NL20 (Supplementary Fig. 1).

#### Functional studies on miR-143 and miR-145 *in vitro*

To investigate the potential function of miR**-**143 and miR**-**145 in NSCLC tumorigenesis, we performed a series of *in vitro* experiments. By transfecting various NSCLC cell lines with miR-143 mimic, miR-145 mimic and miR-143+miR145 mimic, we observed the biomarkers effect on cell migration and proliferation.

#### miR-143 and miR-145 inhibit NSCLC migration

**Figure 2:**
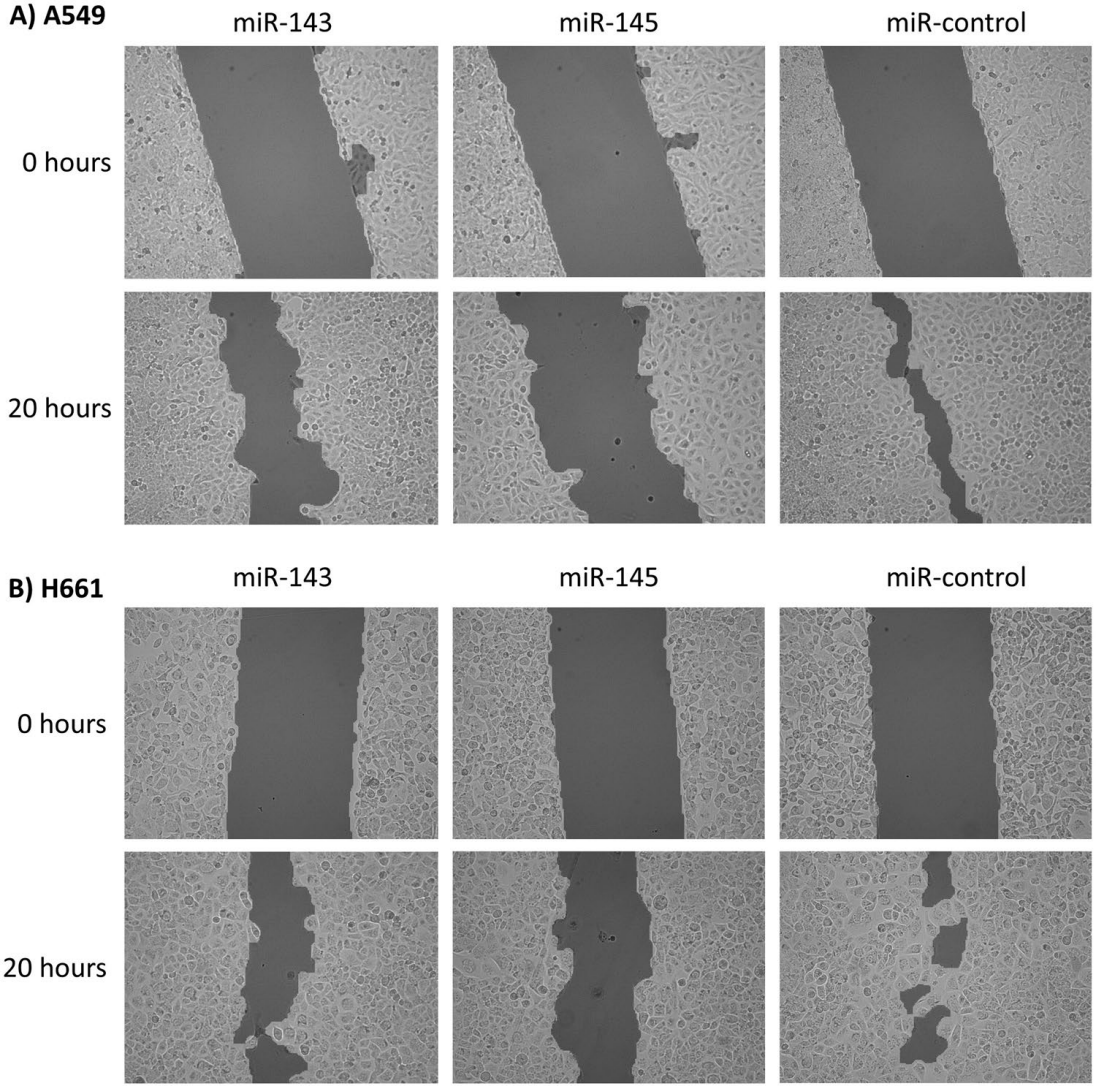
Functional studies on NSCLC cell lines: Migration. (**A**,**B**) Show that migration is inhibited in cells transfected with either miR-143 or miR-145 compared to cells transfected with the scrambled negative control. Data was collected at time point 0 and 20.

Transfection with miR-143 and miR-145 inhibited migration in both the A549 and H661 cell line when compared with cells transfected with the negative control miRNA (Fig. 2). The inhibition was strongest for miR-145 in both cell lines.

#### Inhibition of proliferation by miR-143 and miR-145

**Figure 3:**
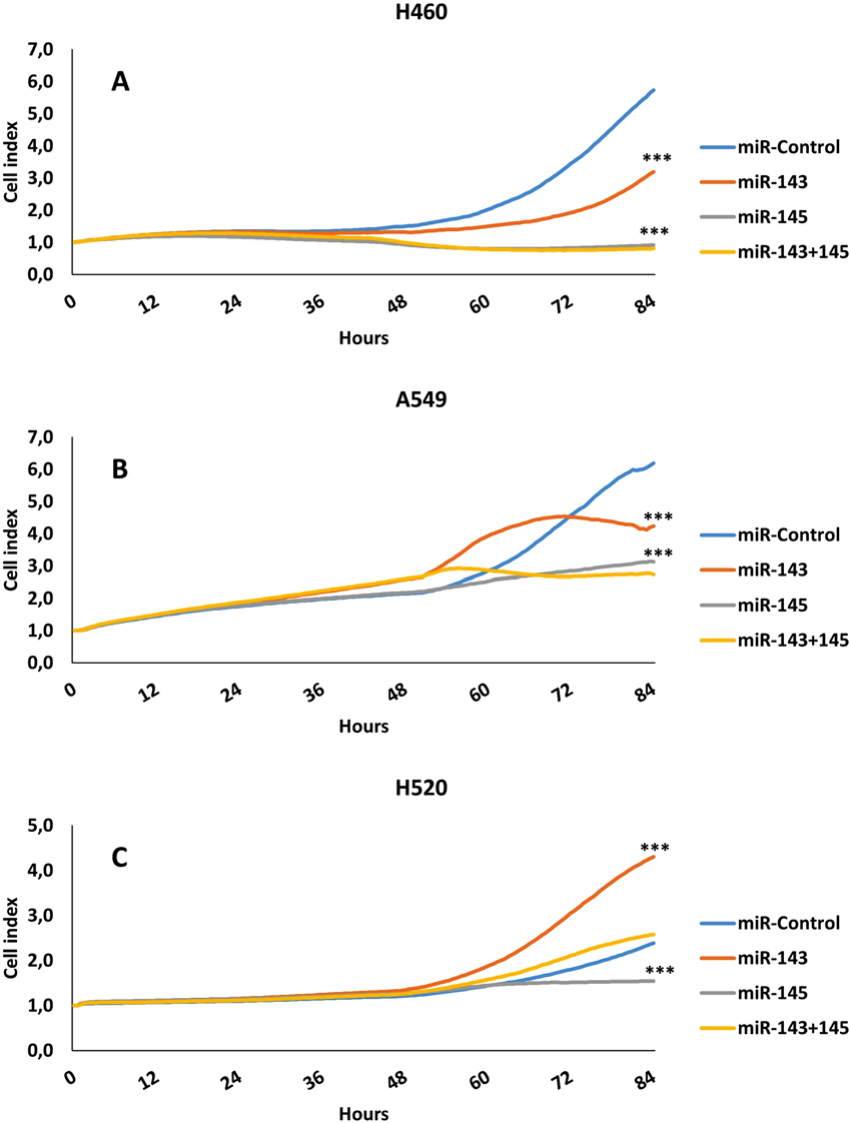
Functional studies on NSCLC cell lines: Proliferation. Panels show proliferation rate in cell lines H460, A549 and H520. Cell Index represents proliferation as a function of time. Panel A-H460: Both miR-143 and miR-145, alone or in combination, inhibits proliferation in H460 cells. The inhibitory effect is strongest in cells receiving the miR-145. Panel B-A549: Same inhibition pattern as panel (A). Panel C-H520: Introducing miR-143 promotes proliferation in this cell line, while transfection with miR-145 inhibits proliferation. *** indicates p < 0.001.

Both miR-143 and miR-145 inhibited proliferation in the cell lines H460 and A549, and the inhibition was more evident for cells transfected with miR-145 (Fig. 3A,B). Transfection of miR-143 promoted proliferation in the H520 cell line, whereas miR-145 had an inhibitory effect on proliferation in the same cell line (Fig. 3C). In the cell lines A549 and H460, the inhibitory effects of co-transfection with miR-143 and miR-145 in equal concentrations, were equivalent to that of the miR-145 transfection alone. When co-transfecting the H520 cell line with equal concentrations of miR-143 and miR-145, the inhibitory effects displayed by transfecting miR-145 alone were reduced to a degree where the proliferation-rate was not significantly different to the negative control. Simultaneously, the increase in proliferation caused by the miR-143 transfection alone, was greatly reduced when the H520 cell line was co-transfected with both miR-143 and miR-145 in equal concentrations.

#### Correlation with clinical variables and other molecular markers

There were no significant associations between miR-143 and miR-145 expression in PT or LN+ and clinicopathological prognosticators listed in Table 1.

Between marker correlations with likely biological significance were as follows: LN+T-miR-143 was positively correlated with PT stromal AR expression (*r* = 0.494: p < 0.001), and inversely correlated with PT tumor epithelial PGR expression (−*r* = 0.453: p < 0.001). T-miR-143 in PT was correlated with cytoplasmic ERβ in PT (*r* = 0.215: p < 0.001) and T-miR-145 in PT was correlated with nuclear ERβ expression in tumor cells (*r* = 0.212: p < 0.001). Other significant correlations were also observed (Supplementary Table 1).

### Univariate survival analyses

**Figure 4:**
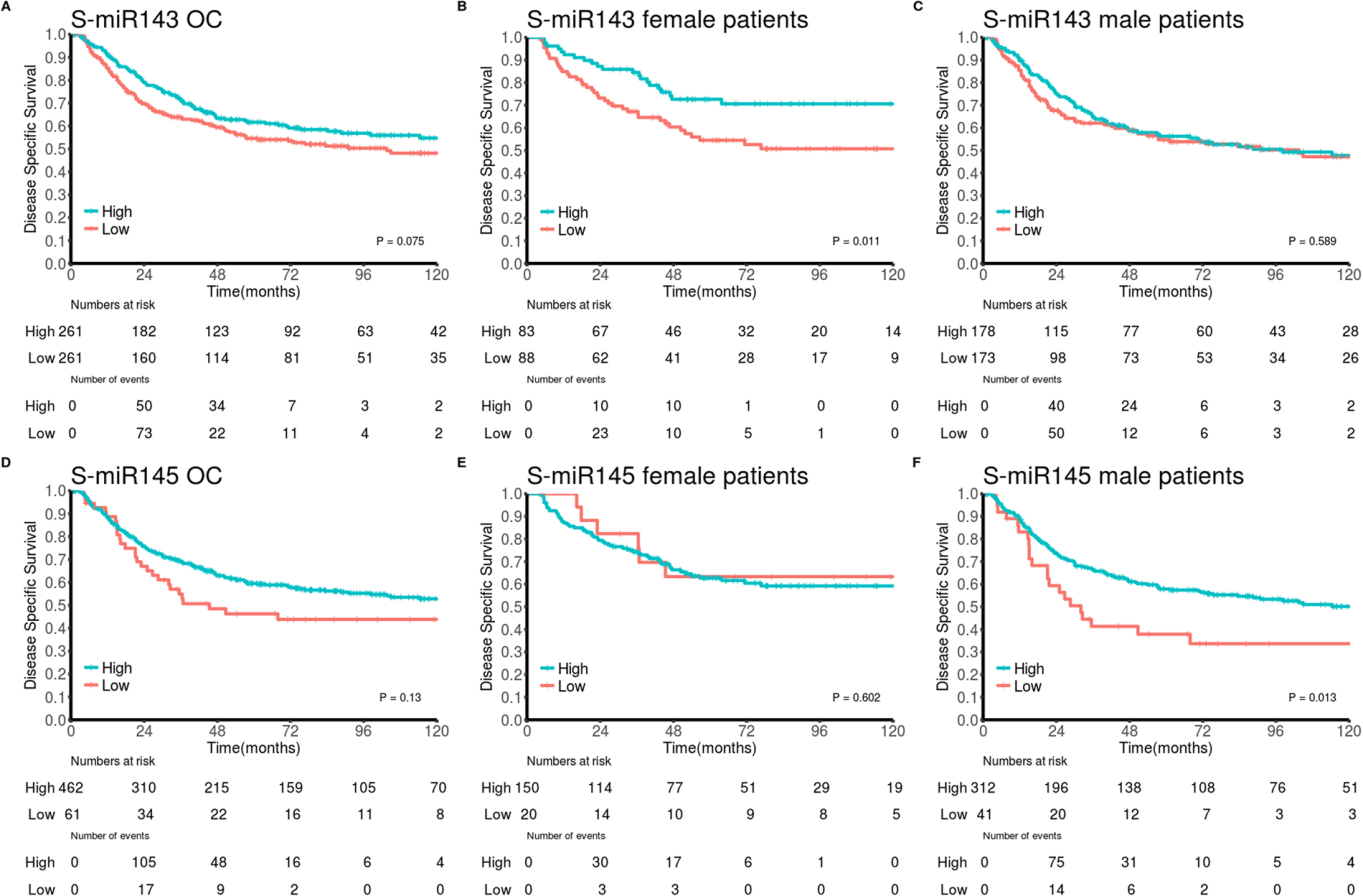
Survival curves. Kaplan-Meier curves showing disease-specific survival (DSS) in relation to stromal miR-143 and miR-145 expression in primary tumor. By dichotomizing biomarker expression level into high vs low, we present an association between improved DSS and high stromal biomarker expression. Overall cohort (OC): panel (A) (miR-143) and panel (D) (miR-145), female patients: panel (B) (miR-143) and panel (E) (miR-145), male patients: panel (C) (miR-143) and panel (F) (miR-145).

**Figure 5:**
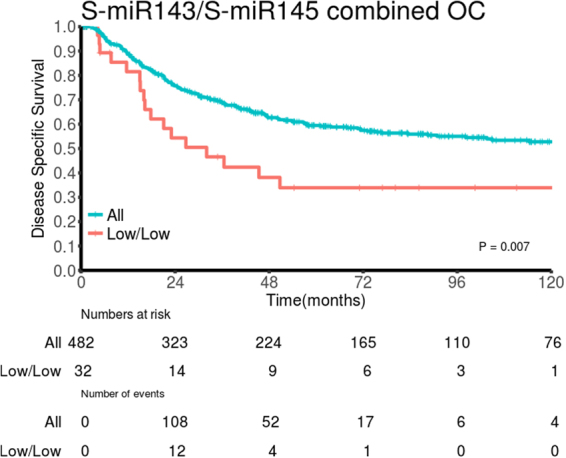
Survival curve. DSS survival curve according to co-expression of stromal miR-143 and stromal miR-145 in primary tumor in the overall cohort (OC). Low/low: low stromal miR-143 expression in combination with low stromal miR-145 expression.

Clinicopathological variables and their impact on DSS are presented in Table 1. The impacts of biomarkers on DSS in PT are presented in Table 2 and Fig. 4. Neither epithelial nor stromal expression of miR-143 or miR-145 showed significant impact on DSS in the overall cohort. Following gender stratification, however, high S-miR-143 was a positive prognosticator in female patients (p = 0.011), while high S-miR-145 was a positive prognosticator in male patients (p = 0.013). Further, the combination of low S-miR-143 and low S-miR-145 was associated with an unfavorable prognosis in the overall cohort (p = 0.007, Fig. 5). In LN+, neither miR-143 nor miR-145 showed impact on DSS in the overall cohort or stratified by gender.

### Multivariate analysis

Significant clinicopathological and biomarker variables from univariate analyses were entered into the multivariate analyses. Results are presented in Table 3. In primary tumors (PT), high S-miR-143 (HR: 0.53, 95% CI: 0.31–0.90, p = 0.019) and high S-miR-145 (HR: 0.58, 95% CI: 0.37–0.92, p = 0.021) were independent, positive prognosticators in female and male patients, respectively. The combination low S-miR-143/low S-miR-145 (overall cohort: HR: 0.57, 95% CI: 0.35–0.94, p = 0.027) was independently associated with an unfavorable DSS.

**Table 3:** Results of Cox regression analysis summarizing significant independent prognostic factors for disease-specific survival (DSS) in primary tumors (PT) in the overall cohort and stratified by gender (N = 553, 180 and 373, respectively).

	Overall cohort	p	Female patients	p	Male patients	p
	HR (95% CI)		HR (95% CI)		HR (95% CI)	
Pstage		<**0**.**001**		<**0**.**001**		<**0**.**001**
I	1 (ref)		1 (ref)		1 (ref)	
II	1.47 (1.03–2.09)	**0**.**033**	1.80 (0.92–3.55)	0.088	1.45 (0.96–2.18)	0.075
IIIA + IIIB	3.82 (2.69–5.44)	<**0**.**001**	5.02 (2.64–9.55)	<**0**.**001**	3.36 (2.28–5.06)	<**0**.**001**
Vascular infiltration		**0**.**001**		**0**.**049**		**0**.**001**
No versus Yes	1.82 (1.30–2.57)		1.75 (1.00–3.06)		2.10 (1.37–3.22)	
Sex		**0**.**010**	NE		NE	
Female versus Male	1.50 (1.10–2.04)					
ECOG perf. status		**0**.**028**	NE			**0**.**050**
0	1 (ref)				1 (ref)	
1	1.45 (1.09–1.93)	**0**.**012**			1.51 (1.08–2.11)	**0**.**017**
2	1.57 (0.82–3.03)	0.176			1.47 (0.71–3.08)	0.301
Primary tumors						
S-miR143				**0**.**019**	NE	
Low vs High	NE		0.53 (0.31–0.90)			
S-miR145	NE					**0**.**021**
Low vs High					0.58 (0.37–0.92)	
S-miR143/S-miR145		**0**.**027**	NE			**0**.**027**
Low vs High	0.57 (0.35–0.94)				0.50 (0.27–0.92)	

Abbreviations: S-miR, stromal miR expression. CI, confidence interval. ECOG perf. status, Eastern Cooperative Oncology Group performance status. HR, Hazard ratio. NE, not entered.

## Discussion

In this large retrospective study of 553 NSCLC patients, S-miR-143 and S-miR-145 expression in PT were positive prognosticators in female and male patients, respectively. Further, the combination of low stromal expression of both miR-143 and miR-145 predicted poor DSS in the overall cohort. Cell line studies confirm the tumor suppressive role of miR-143 and miR-145 in NSCLC, further substantiating their importance in lung cancer pathogenesis. We also observe significant correlations with our previously investigated steroid hormone receptors, suggesting that a biologic rationale may cause, or contribute to, the gender related survival impact observed.

To our knowledge, this is the first study investigating the prognostic impact of miR-143 and miR-145 in neoplastic epithelial cells, tumor associated stromal cells and matched metastatic lymph nodes in the same NSCLC cohort.

Associations between miR cluster 143/145 and cancer survival have been reported for different malignancies, results are, however, conflicting. In prostate cancer (PCa), Avgeris *et al*.^17^ demonstrated a shorter disease-free survival in PCa patients with low miR-145 expression levels. Campayo *et al*.^18^ reported similar results for miR-145 in NSCLC patients. These reports confirm our suggestion of high miR-145 expression as a positive prognosticator, herein in NSCLC patients. Contradicting our results, Al Feber *et al*.^19^ and Avgeris *et al*.^20^, both reported associations between high levels of miR-143 and miR-145 and poor overall survival in esophageal and bladder cancer, respectively. Importantly, none of the aforementioned studies have evaluated survival impact according to cellular compartment, as was performed in our study.

Our findings suggest miR-143 and miR-145 to play protective roles when expressed in stromal cells. This is in concordance with the biomarkers being regarded as tumor suppressors^21^. miR-143 and miR-145 target several important genes involved in tumorigenesis including KRAS^22^ and ERα^11^. However, their biological function in NSCLC remains largely unknown. Functional studies have proposed different roles; Chen *et al*., 2010 demonstrated that miR-145 inhibited NSCLC proliferation by targeting the transcription factor regulatory gene *c-Myc*^13^, thus confirming the tumor suppressive qualities of this particular miR. In a recent report Zhang *et al*.^12^ suggest epidermal growth factor receptor (EGFR) as a downstream target of miR-143, contributing to tumor suppression. Consistent with our findings, Zhang *et al*.^12^ demonstrated an inhibition of migration and proliferation of NSCLC cells following transfection with miR-143. Surprisingly, when we transfected the squamous cell carcinoma cell line H520 with miR-143, proliferation was dramatically increased (Fig. 3C). This is in contrast to most studies reporting on the effects of miR-143 on proliferation^23–27^. However, there are studies depicting alternative roles for the miR cluster 143/145^28^, and members of our research group have reported similar findings using breast cancer cell lines^29^. Interestingly, when co-transfecting miR-143 and miR-145 in equal concentrations, the proliferative capacity was markedly reduced (Fig. 3A,B), meaning the net effect of co-transfecting is tumor suppressive.

The use of verified laboratory techniques with meticulously prepared protocols for biomarker handling is a strength with regards to reliability and reproducibility of our results. Our patient cohort is large with an extensive follow-up time, which further substantiate our results. The retrospective study design may represent a weakness with inaccurate clinical patient data.

In line with previous reports, we detect downregulation of both miR-143 and miR-145 in four independent cancer cell lines relative to levels in a non-cancerous cell line (Supplementary Fig. 1)^30,31^. Thus, extending the general sense of miR-143/miR-145 downregulation in cancer, including NSCLC^21^. Interestingly, this is in contrast with our ISH-results, reporting significantly increased expression of miR-143/miR-145 in tumor cells and adjoining stromal cells in comparison to non-malignant tissue. To our knowledge, this is the first large-scale miRNA *in situ* NSCLC tissue hybridization analysis reporting an upregulated miR-143/miR-145 expression. These findings are conflicting with the smaller study (n = 48) by Shen *et al*.^31^, reporting a downregulation of miR-145 expression in NSCLC, by the use of ISH technique. We present a thorough and comprehensive study of miR-143 and miR-145 expression in appropriate cell types by the use of several acknowledged techniques, giving an optimal account of miR-expression in the tumor environment. Due to contributions from stromal cells in tumor growth, it is pivotal to consider the stromal compartment when elucidating biological mechanisms in epithelial cancers^32^. We found that miR-143 was primarily observed in the cell cytoplasm, while miR-145 was mainly observed in the nuclei of epithelial and stromal cells. Further, we report an abundant positivity of miR-143 and miR-145 in fibroblasts and SMCs lining the blood and lymph vessels, consistent with previous reports^9,33^. miRs are traditionally considered to act within the cell cytoplasm, regulating gene expression post-transcriptionally^34^. However, a number of miRNAs have been localized in the nuclei, although their nuclear functions remain elusive^35^.

In an extensive meta-analysis, Kent *et al*.^9^ highlighted the crucial importance of cell-type localization of miRNAs, and how a lack of consideration of specific cellular expression of miRNAs may lead to a general misconception that miRNAs are downregulated in neoplastic tissue. Chivukula *et al*.^36^, Dimitrova *et al*.^28^ and Akao *et al*.^37^ all published results indicating that neither miR-143 nor miR-145 are expressed in tumor epithelial cells, causing the latter group to conclude that these miRNAs are downregulated in malignant tissue. Similar results have been published by other groups investigating a variety of malignancies^21,38,39^. However, only one^28^ of the previous studies has focused on the histological cell-type localization of miR-143/miR-145. These factors, combined with the lung cancer cell lines lack of stroma, inflammatory cells and vascularization, may contribute to the discrepancy observed between miR-143/miR-145 expression in cell lines and tissue samples.

In this study, we present interesting correlations between miR-143/miR-145 and steroid hormone receptors expressed in the NSCLC tissue. The finding of gender specific survival significance of miR-143 and miR-145, forces us to consider sex hormones as a relevant factor. Delfino *et al*.^40^ reported a gender-specific miRNA targeting of molecules related to glioblastoma survival. Further, Duttagupta *et al*.^41^ reported differential miRNA expression levels in a gender specific manner. Mounting evidence confirms activation of hormone receptors to be of outmost importance in lung cancer pathogenesis and several interesting cross-talk pathways between steroid hormones and miR-143/miR-145 have been found^42–46^. Both miRNAs play a critical role in ovarian functioning, and a recent report presents miR-143 affecting estradiol production in granulosa cells by targeting KRAS^47,48^. Further, Spizzo *et al*., 2011 reported that miR-145 downregulates ERα expression in breast cancer^11^. We found correlations between miR-143/miR-145 and AR, the rate limiting enzyme in estradiol production, suggesting the miRs may interact with regional estradiol production and ER signaling in the lung, as observed in breast tissue. In 2012, Paris *et al*. presented a study on estrogen effects in breast cancer, showing a direct regulation of miRNA expression and ERβ signaling^49^. Herein, ERβ expression correlated with miR-143/miR-145 expression, suggesting a similar link may exist in NSCLC. The aforementioned reports, assembled with our findings, provide a compelling rationale for a biological cross-talk between miR-143/miR-145 and hormone receptors. If validated in larger, confirmatory studies, this may in fact represent new possibilities for targeted therapy for NSCLC patients, using gender, miRNA and hormone receptor expression as therapy selection criteria.

## Conclusion

We present high stromal expressions of miR-143 and miR-145 as positive prognosticators in a gender specific manner in early stage NSCLC patients. Our findings indicate that miR-143/miR-145 acts as tumor suppressor molecules in lung cancer, suggesting that these miRNAs may be useful in miRNA based therapy in NSCLC. Further, we highlight the complexity of miR expression, and stress the importance of cell-type specific expression profiling. By accentuating the correlation between miRNA expression and hormone receptor expression, we emphasize the importance of exploring multi-targeted therapies in the treatment of NSCLC patients, as anti-hormonal therapy is highly accessible.

## Electronic supplementary material

Supplementary information

## References

[1] Ferlay, J. *et al*. Cancer incidence and mortality worldwide: sources, methods and major patterns in GLOBOCAN 2012. *International journal of cancer* **136** (2015). 10.1002/ijc.29210 25220842

[2] Travis, W. D., Brambilla, E., Burke, A., Marx, A. & Nicholson, A. G. *WHO classification of tumours of the lung*, *pleura*, *thymus and heart*. (International Agency for Research on Cancer, 2015). 10.1097/JTO.0000000000000663 26291007

[3] Siegel RL, Miller KD, Jemal A (2016). Cancer statistics, 2016. CA: a cancer journal for clinicians.

[4] Bartel DP (2004). MicroRNAs: genomics, biogenesis, mechanism, and function. Cell.

[5] Doench JG, Sharp PA (2004). Specificity of microRNA target selection in translational repression. Genes & development.

[6] Calin GA, Croce CM (2006). MicroRNA signatures in human cancers..

[7] Wiggins JF (2010). Development of a lung cancer therapeutic based on the tumor suppressor microRNA-34. Cancer research.

[8] Reid G (2016). Clinical development of TargomiRs, a miRNA mimic-based treatment for patients with recurrent thoracic cancer. Epigenomics.

[9] Kent OA, McCall MN, Cornish TC, Halushka MK (2014). Lessons from miR-143/145: the importance of cell-type localization of miRNAs. Nucleic acids research.

[10] Zhang J (2013). Loss of microRNA-143/145 disturbs cellular growth and apoptosis of human epithelial cancers by impairing the MDM2-p53 feedback loop. Oncogene.

[11] Spizzo R (2010). miR-145 participates with TP53 in a death-promoting regulatory loop and targets estrogen receptor-α in human breast cancer cells. Cell death and differentiation.

[12] Zhang HB, Sun LC, Ling L, Cong LH, Lian R (2016). miR-143 suppresses the proliferation of NSCLC cells by inhibiting the epidermal growth factor receptor. Experimental and Therapeutic Medicine.

[13] Chen Z (2010). miRNA-145 inhibits non-small cell lung cancer cell proliferation by targeting c-Myc. Journal of Experimental & Clinical Cancer Research.

[14] Goldstraw P (2016). The IASLC Lung Cancer Staging Project: proposals for revision of the TNM stage groupings in the forthcoming (eighth) edition of the TNM classification for lung cancer. Journal of Thoracic Oncology.

[15] Bremnes R (2002). High-throughput tissue microarray analysis used to evaluate biology and prognostic significance of the E-cadherin pathway in non–small-cell lung cancer. Journal of Clinical Oncology.

[16] McShane LM (2005). REporting recommendations for tumour MARKer prognostic studies (REMARK). British journal of cancer.

[17] Avgeris M, Stravodimos K, Fragoulis E, Scorilas A (2013). The loss of the tumour-suppressor miR-145 results in the shorter disease-free survival of prostate cancer patients. British journal of cancer.

[18] Campayo M (2013). Low miR-145 and high miR-367 are associated with unfavourable prognosis in resected nonsmall cell lung cancer. European Respiratory Journal.

[19] Feber A (2011). MicroRNA prognostic signature for nodal metastases and survival in esophageal adenocarcinoma. The Annals of thoracic surgery.

[20] Avgeris M (2015). Uncovering the clinical utility of miR-143, miR-145 and miR-224 for predicting the survival of bladder cancer patients following treatment. Carcinogenesis.

[21] Das AV, Pillai RM (2015). Implications of miR cluster 143/145 as universal anti-oncomiRs and their dysregulation during tumorigenesis. Cancer cell international.

[22] Kent OA (2010). Repression of the miR-143/145 cluster by oncogenic Ras initiates a tumor-promoting feed-forward pathway. Genes & development.

[23] Wang Q, Cai J, Wang J, Xiong C, Zhao J (2014). MiR-143 inhibits EGFR-signaling-dependent osteosarcoma invasion. Tumour Biol.

[24] Xia H (2014). miR-143 inhibits NSCLC cell growth and metastasis by targeting Limk1. Int J Mol Sci.

[25] Liu J (2016). MiR-143 inhibits tumor cell proliferation and invasion by targeting STAT3 in esophageal squamous cell carcinoma. Cancer letters.

[26] Xu YF (2015). Identification of miR-143 as a tumour suppressor in nasopharyngeal carcinoma based on microRNA expression profiling. Int J Biochem Cell Biol.

[27] Zhang W, Wang Q, Yu M, Wu N, Wang H (2014). MicroRNA-145 function as a cell growth repressor by directly targeting c-Myc in human ovarian cancer. Technology in cancer research & treatment.

[28] Dimitrova N (2016). Stromal Expression of miR-143/145 Promotes Neoangiogenesis in Lung Cancer Development. Cancer Discov.

[29] Johannessen C (2017). Expression and function of the miR-143/145 cluster *in vitro* and *in vivo* in human breast cancer. PLoS One.

[30] Takagi T (2009). Decreased expression of microRNA-143 and-145 in human gastric cancers. Oncology.

[31] Shen H (2015). Low miR-145 expression level is associated with poor pathological differentiation and poor prognosis in non-small cell lung cancer. Biomedicine & Pharmacotherapy.

[32] Almeida MI, Calin GA (2016). The miR-143/miR-145 cluster and the tumor microenvironment: unexpected roles. Genome medicine.

[33] Cordes KR (2009). miR-145 and miR-143 regulate smooth muscle cell fate decisions. Nature.

[34] Roberts TC (2014). The MicroRNA Biology of the Mammalian Nucleus. Molecular therapy. Nucleic acids.

[35] Liao JY (2010). Deep sequencing of human nuclear and cytoplasmic small RNAs reveals an unexpectedly complex subcellular distribution of miRNAs and tRNA 3’ trailers. PloS one.

[36] Chivukula RR (2014). An essential mesenchymal function for miR-143/145 in intestinal epithelial regeneration. Cell.

[37] Akao Y, Nakagawa Y, Naoe T (2006). MicroRNAs 143 and 145 are possible common onco-microRNAs in human cancers. Oncology reports.

[38] Lui W-O, Pourmand N, Patterson BK, Fire A (2007). Patterns of known and novel small RNAs in human cervical cancer. Cancer research.

[39] Szczyrba J (2010). The microRNA profile of prostate carcinoma obtained by deep sequencing. Molecular cancer research.

[40] Delfino K, Serão N, Southey B, Rodriguez-Zas S (2011). Therapy-, gender-and race-specific microRNA markers, target genes and networks related to glioblastoma recurrence and survival. Cancer Genomics-Proteomics.

[41] Duttagupta R, Jiang R, Gollub J, Getts RC, Jones KW (2011). Impact of cellular miRNAs on circulating miRNA biomarker signatures. PloS one.

[42] Weinberg OK (2005). Aromatase inhibitors in human lung cancer therapy. Cancer research.

[43] Mah V (2007). Aromatase expression predicts survival in women with early-stage non–small cell lung cancer. Cancer research.

[44] Márquez-Garbán DC, Chen H-W, Fishbein MC, Goodglick L, Pietras RJ (2007). Estrogen receptor signaling pathways in human non-small cell lung cancer. Steroids.

[45] Skjefstad K (2016). Prognostic relevance of estrogen receptor α, β and aromatase expression in non-small cell lung cancer. Steroids.

[46] Skjefstad K (2015). The prognostic role of progesterone receptor expression in non-small cell lung cancer patients: Gender-related impacts and correlation with disease-specific survival. Steroids.

[47] Zhang L (2017). MiRNA-143 mediates the proliferative signaling pathway of FSH and regulates estradiol production. Journal of Endocrinology.

[48] Hossain M, Sohel M, Schellander K, Tesfaye D (2012). Characterization and importance of microRNAs in mammalian gonadal functions. Cell and tissue research.

[49] Paris O (2012). Direct regulation of microRNA biogenesis and expression by estrogen receptor beta in hormone-responsive breast cancer. Oncogene.

