# Supplementary material for: A gender specific improved survival related to stromal miR-143 and miR-145 expression in non-small cell lung cancer
Source: Sci Rep. 2018 Jun 4;8:8549. doi: 10.1038/s41598-018-26864-w (PMC5986811; doi:10.1038/s41598-018-26864-w)
Supplement: Supplementary file 1 — Supplementary information [file 41598_2018_26864_MOESM1_ESM.pdf]

A gender specific improved survival related to stromal miR-143 and miR-145 expression in non-small cell lung cancer

Kaja Skjefstad, MD[1]*
Charles Johannessen, MSc[1]
Thea Grindstad, MD[1]
Thomas Kilvaer, MD, PhD[2,3]
Erna-Elise Paulsen, MD, PhD[2,3]
Mona Pedersen[2]
Tom Donnem, MD, PhD[2,3]
Sigve Andersen, MD, PhD[2,3]
Roy Bremnes, MD, PhD[2,3]
Elin Richardsen, MD, PhD[1,4]
Samer Al-Saad, MD, PhD[1,4]
Lill-Tove Busund, MD, PhD[1,4]

[1] Department of Medical Biology, UiT The Arctic University of Norway, Mailbox 6050 Langnes, N-9037 Tromso, Norway
[2] Department of Clinical Medicine, UiT The Arctic University of Norway, Mailbox 6050 Langnes, N-9037 Tromso, Norway
[3] Department of Oncology, University Hospital of North Norway, Mailbox 13, N-9038 Tromso, Norway
[4] Department of Clinical Pathology, University Hospital of North Norway, Mailbox 46, N-9038 Tromso, Norway

E-mail addresses in order as listed above: kaja.skjefstad@gmail.com, charles.johannessen@uit.no, theagrindstad@gmail.com, kilvaer@gmail.com, erna.e.paulsen@uit.no, mona.i.pedersen@uit.no, tom.donnem@uit.no, sigve.andersen@uit.no, roy.bremnes@uit.no, elin.richardsen@unn.no, sameralsaad@gmail.com, lill.tove.busund@unn.no

*** Corresponding author**
Kaja Konstanse Skjefstad, MD
Translational Cancer Research Group
Department of Medical Biology
UiT The Arctic University of Norway
Mailbox 6050 Langnes
9038 Tromso, Norway
Telephone: +47 48 14 04 06
E-mail: kaja.skjefstad@gmail.com

Supplementary Table 1
Spearman rank correlations between mean scores of tumor epithelial (T-) and stromal (S-) miR-143 and miR-145 in primary tumor (PT) and metastatic lymph nodes (LN+) and other molecular markers

	PT S-miR-143	PT T-miR-143	LN-miR-143	PT S-miR-145	PT T-miR-145	LN-miR-145
S-AR			0.494***			
ERβ_cyt		0.215***				
ERβ_nuc	-0.125*				0.212***	
S-PGR			-0.336*			
T-PGR			-0.453***			
T-PDGFA			0.287*			
S-bFGF			0.288*			
S-FGFR1			0.318*			
S-Notch4			0.289*			
T-Ang1		0.209***				
T-Tie2					0.223**	
T-MCT1		0.297***				
T-MCT4			-0.306*			
T-Thro					0.212**	
T-B_celle						0.312*
T-pTEN			-0.320*			
T-pHer				0.216***		
T-Her3		0.226***				0.296*
S-vim						-0.334*
T-APK					0.215***	
tFC					0.232***	
T-Igfbp						0.298*
S-Igf			-0.268*			
pFC					0.299***	
pFN			-0.290*			
MET_g			-0.354*			
S-Ki67			-0.285*			

* = $p<0.05$, ** = $p<0.01$, *** = $p<0.001$

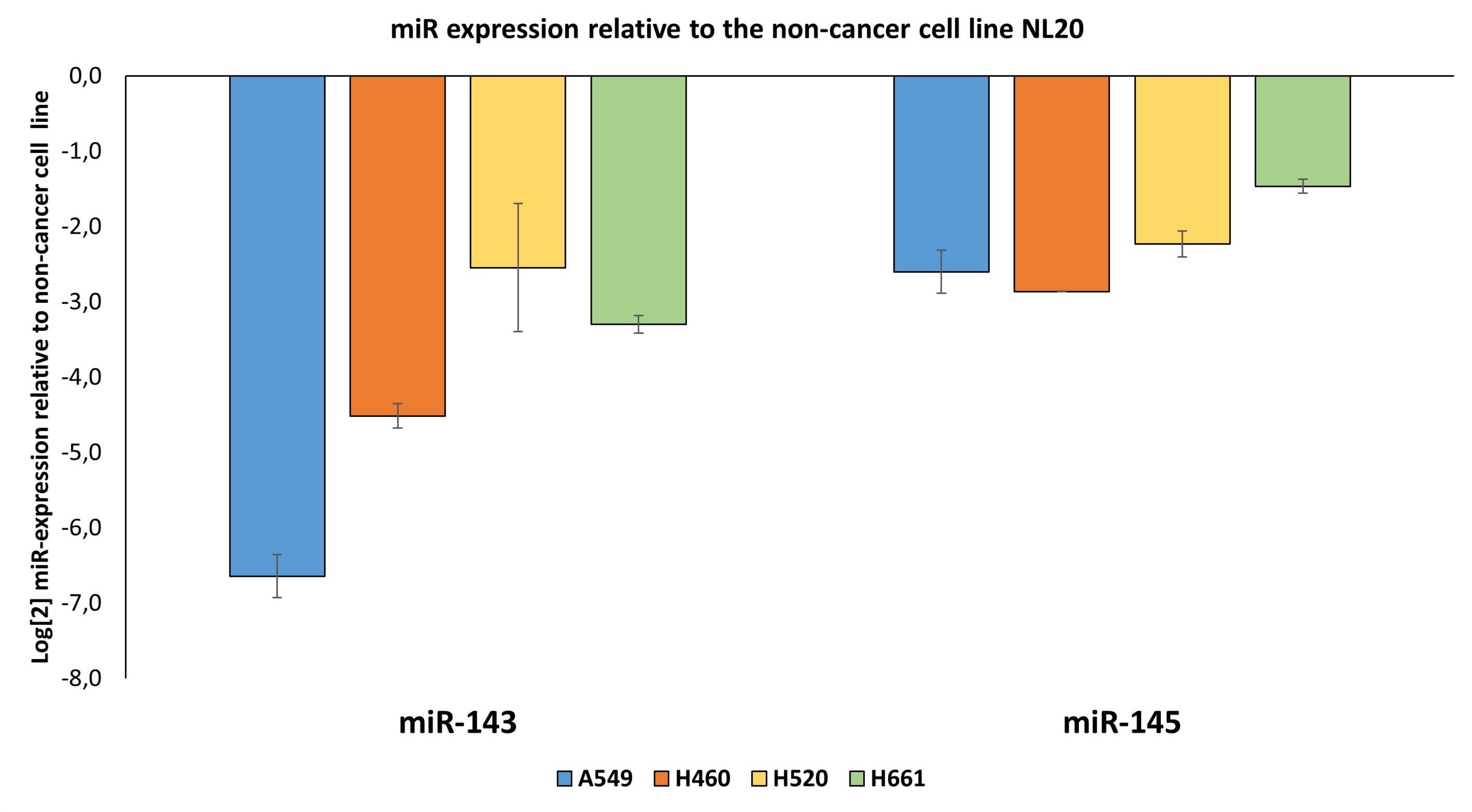
miR expression relative to the non-cancer cell line NL20
Log[2] miR-expression relative to non-cancer cell line
0,0
-1,0
-2,0
-3,0
-4,0
-5,0
-6,0
-7,0
-8,0
miR-143
miR-145
A549
H460
H520
H661

Legend to supplementary figure

Supplementary figure 1

Endogenous levels of miR-143 and miR-145 in the studied NSCLC cell lines were quantified by qPCR, relative to the non-cancerous lung cell line NL20. Both miR-143 and miR-145 were downregulated in all the selected cell lines, compared to NL20
